# Study of Human Tacit Knowledge Based on Electroencephalogram Signal Characteristics

**DOI:** 10.3389/fnins.2021.690633

**Published:** 2021-07-14

**Authors:** Tao Zhang, Chengcheng Hua, Jichi Chen, Enqiu He, Hong Wang

**Affiliations:** ^1^Department of Mechanical Engineering and Automation, Northeastern University, Shenyang, China; ^2^College of Applied Technology, Shenyang University, Shenyang, China; ^3^School of Mechanical Engineering, Shenyang University of Technology, Shenyang, China

**Keywords:** tacit knowledge, electroencephalogram, industrial process, functional brain network, graph theory

## Abstract

Tacit knowledge is the kind of knowledge that is difficult to transfer to another person by means of writing it down or verbalizing it. In the mineral grinding process, the proficiency of the operators depends on the tacit knowledge gained from their experience and training rather than on knowledge learned from a handbook. This article proposed a method combining the electroencephalogram (EEG) signals and the industrial process to detect the proficiency of the operators in the mineral grinding process to reveal the effect of tacit knowledge on the functional cortical connection. The functional brain networks of operators were established based on partial direct coherence and directed transfer function of EEG, and the multi-classifiers were used with the graph-theoretic indexes of the FBNs as input to distinguish the trained operators (Hps) from the non-trained operators (Lps). The results showed that the brain networks of Hps had a better connectivity than those of Lps (*p* < 0.01), and the accuracy of classification was up to 94.2%. Our studies confirm that based on the performance of EEG features and the combination of industrial operational operation and cognitive processes, the proficiency of the operators can be detected.

## Introduction

Tacit knowledge is the opposite of explicit knowledge. It is a kind of knowledge that is difficult to express to others by writing or using words (Schmidt and Hunter, 1993). In this study, it is simply practical intelligence; for example, the practical intelligence on the operation of the mineral grinding process can be obtained in training, rather than in some handbooks, guidelines, or lectures (Cianciolo et al., 2006; Patalasmaliszewska and Sławomir, 2018). The training significantly improves the generation of tacit knowledge while modulating the neural structure or function of the brain. Therefore, the neural activity can be used as a measure of the tacit knowledge (Cianciolo et al., 2006; Guo et al., 2017).

There was a lot of research on the correlation between tacit knowledge and the neural signatures: Sanchez-Lopez et al. (2016) applied event-related potentials (ERPs) to investigate the difference between the skilled and novice martial arts athletes during their motion-related tasks, especially in the P100 and P200 components. Silva et al., used many assessments including electroencephalogram (EEG) to verify the professionalism of the cyber security teams (Sanchez-Lopez et al., 2016). Gallicchio et al. (2016) investigated the difference between expert golfers and novice golfers in the clustering state among the electrodes in the high alpha rhythm and found that the functional connection between left temporal and frontal regions was related to the performance of the golfers: the connectivity of T7-Fz of the expert golfers was weaker than that of the novice experts. Muraskin et al. (2015) analyzed the difference in EEG when the expert baseball players and the non-expert baseball players estimated the opportunity of swing and found not only that the neural activity in the supplementary movement area of the expert baseball players was more inhibitory than that of the non-expert baseball players but also that the sensor–motor coupling of the expert baseball players was stronger than that of the non-expert baseball players. Maddox et al. (2015) considered the gamma and alpha rhythm as the indexes of the concentration and pressure to estimate the proficiency of the surgeons in the laparoscopic surgical simulation. The results suggested that the concentration and pressure of the expert surgeons were higher and lower, respectively. Luchsinger et al. (2016) found the difference in neural activity in frontal theta rhythm between the expert and the novice athletes. It is noticeable that all these methods of studying neural activity are EEG. The reason why EEG is chosen as a measure of neural activity corresponding to tacit knowledge is that EEG is a relatively low-cost device that can record the subject’s EEG during the task without any interruption. In addition, EEG also provides a higher time solution to help analyze changes in dynamic brain networks over time (Martis, 2013).

However, research on the relationship between the tacit knowledge in the industrial field and the neural activity was few. The tacit knowledge of human beings plays an essential role in most industrial processes. Therefore, the purpose of this study is to reveal the unknown connection between the industrial process and the brain network hidden under the operation training and to detect the operator’s proficiency by analyzing the operator’s functional brain network (FBN) based on the partial directed coherence (PDC) and the directed transfer function (DTF) (Sameshima and Baccalá, 1999; Schelter, 2006; Schelter et al., 2009; Florin et al., 2011). In addition, studying the differences in the functional connection patterns of the various brain regions of the high-proficiency operators and low-proficiency operators at work will help to reveal the relationship between human brain knowledge acquisition and brain function, so as to understand the process of human brain cognition, memory, and information processing. In terms of human–computer interaction, the introduction of cognitive features can help humans evaluate the proficiency of operators more objectively and in advance.

In this study, PDC/DTF methods are employed to reveal whether and how the structures are functionally connected, thereby implying the difference in cognitive ability. In this study, brain network is used to study the changes in the brain function of the operators during work to reveal the internal information flow of the operators’ brain, which helps to understand the cognitive state of the operator in the process of controlling grinding. Our studies confirm that based on the performance of EEG features and the combination of industrial operational operation and cognitive processes, the proficiency of the operators can be detected.

## Materials and Methods

### Experimental Procedure

**FIGURE 1:**
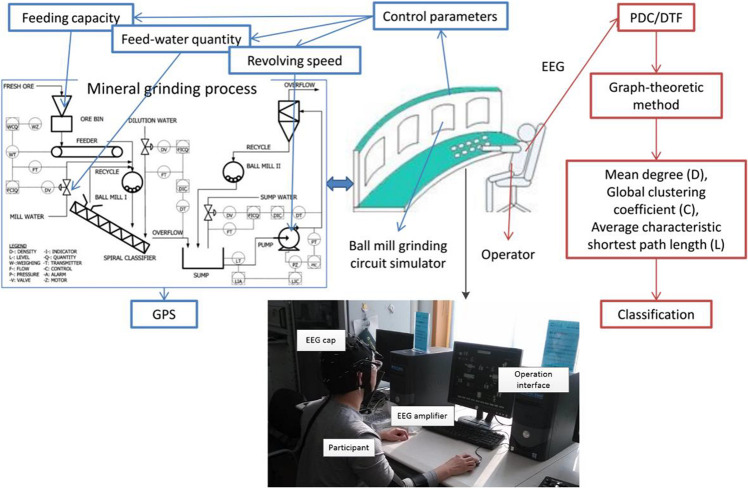
The object is an operator of the mineral grinding process. His/her EEG is recorded while he/she is operating the ball mill grinding circuit stimulator platform. Then, the FBN features are extracted from the EEG to detect the proficiency of the operator.

The experiment was conducted in the State Key Laboratory of Process Industry Automation of China, using a simulation platform developed by Lu et al. (2014). The experiment process is that the operators control the value of three grinding variables to achieve a target grinding particle size (GPS) on the ball mill grinding circuit simulator (see Figure 1). The three variables that the operator should control are the feeding capacity, feed-water quantity, and revolving speed of underflow pump (see Figure 1: control parameters). It is a simulator platform for industrial ball milling control, which can realize the interference and control of process variables and product particle size distribution.

### Subjects and Training

There were two groups of subjects: high-proficiency operators (Hps) and low-proficiency operators (Lps), the total number of which was 20. Each group had 10 operators (Hps: 1 female and 9 males; Lps: 2 females and 8 males). All subjects are volunteer students (mean age: 24 ± 2 years), are right-handed, and have no reported history of neurological disease, neuropsychological problems, or medication and drug abuse. All gave written informed consent to participate in the study. In the experiment, the mineral grinding platform was an open-loop control system, which means that the operators did not monitor the grinding particle size and did not know the effect of their operation. The operational stage introduction of the simulation platform to the two groups is the same. Hps were trained to operate the simulation platform more than once a day (except weekends) for 1 month, and each training time is 0.5–1 h. They would learn more about the details of controlling process from the three different values, such as the degree of influence on GPS, and have the ability to estimate the value of GPS. Each operator had only one chance to control the GPS to the target value during the 5-min experiment. After the training, a 10-point self-evaluation report (1 = low proficiency and 10 = high proficiency) indicated that Hps were successfully identified under the experimental design because their scores were higher than 5 points.

### EEG Recording and Preprocessing

In each trial, the subject is seated on a comfortable chair located about 1 m in front of the computer screen. Each trial run lasted for 5 min, and the subjects should try their best to achieve the target GPS. The EEG of each subject was recorded when he/she was controlling the mineral grinding process though Neuroscan. The cap of Neuroscan has 37 electrodes (30 EEG electrodes were distributed according to the International 10–20 locations plus 4 EOG reference electrodes; 2 reference electrodes were placed on the mastoid behind the ears, and 1 GND electrode was on the forehead) based on saline sensors. The sampling rate is 1,000 Hz. The electrode impedance was decreased by using saline liquid until the level required by the software was reached (in the 10–20 kΩ range) and was checked along the experiment.

**FIGURE 2:**
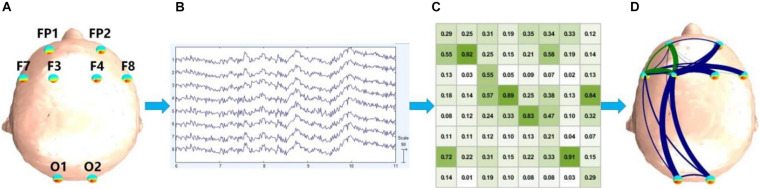
General block diagram of the proposed system. **(A)** Electrode position; **(B)** EEG signal, where the horizontal axis represents time and the vertical axis represents electrodes; **(C)** adjacent matrix, and the values in each cell represent PDC or DTF we calculated; **(D)** functional brain network.

The vision and execution are the key functions involved in the mineral grinding task, which mainly refer to the prefrontal and occipital regions of the cortex (Zeki et al., 1991; Duncan and Owen, 2000), so in our experiment, only the eight EEG electrodes FP1, FP2, F7, F8, F3, F4, O1, and O2 were used (Figure 2A). The collected EEG signal is shown in Figure 2B. All signals were low-pass filtered (cutoff frequency 50 Hz) and then downsampled to 128 Hz to reduce the data size. For the following calculation and classification, about 180 epochs with 1.5 s non-overlap were segmented and extracted uniformly from each subject. Each epoch lasts for 1,280 points (10 s). Then, 3,343 epochs of EEG data were obtained to build FBNs.

### Functional Brain Networks

**FIGURE 3:**
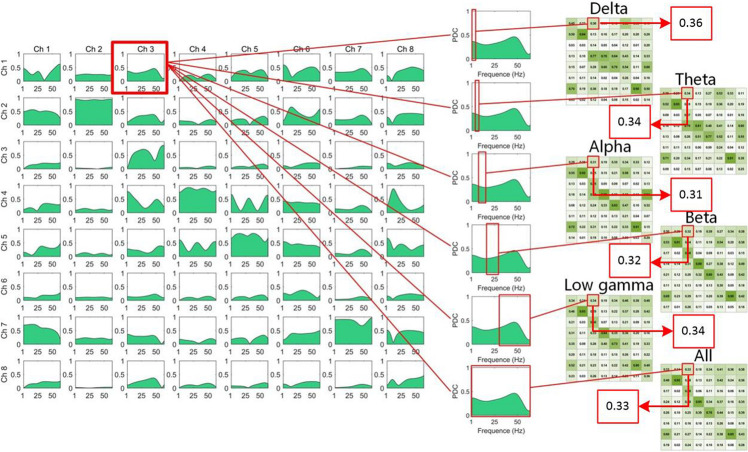
The computation of the adjacent matrices from the PDC/DTF in the different frequency bands. The matrices of PDC or DTF we calculated were 3D [π*_*ij*_* (*f*) and *v*_*ij*_ (*f*) were the elements of the 3D PDC and the DTF matrix, respectively]. The two Ds of PDC or DTF matrix were channels [*i* and *j*: FP1 (Ch1), FP2 (Ch2), F7 (Ch3), F8 (Ch4), F3 (Ch5), F4 (Ch6), O1 (Ch7), and O2 (Ch8)], and the third D was f: frequency (0–64 Hz). We divided the third D into the following ranges: Delta (δ): 0.5–4.0 Hz, Theta (θ): 4.0–8.0 Hz, Alpha (α): 8.0–14 Hz, Beta (β): 15.0–30.0 Hz, Low gamma (γ): 30.5–50 Hz, All: 0–50 Hz, and then computed the mean value of functional connectivity in different ranges. Hence, the six matrices of different frequency ranges were computed and plotted in the right of the figure. Due to the different values between the two channels, eight channels were represented twice, as row and column headings.

The construction of the FBN is shown in Figure 3. There are advantages to proposing FBN methods in the research of the EEG, because the ability of the brain to conduct high-level sensory and cognitive functions depends strongly on underlying interactions between two different brain functional regions (Bullmore and Sporns, 2009). Although the analysis of structural networks helps us to understand the fundamental architecture of inter-regional connections, the functional networks must be directly considered to elucidate how this architecture supports neuro-physiological dynamics (Park and Friston, 2013; Coullaut-Valera et al., 2014). The PDC/DTF methods are popular in the analysis of the functional connection based on EEG signals in recent years, and they are parametric methods to estimate the causality among multi-channel signals with the consideration of all channel signals’ influence. The two methods were named by Kaminski and Blinowska (1991) and Baccala et al. (2001), respectively. They can obtain more information than other popular methods such as synchronization likelihood (Stam and Van Dijk, 2002), fuzzy synchronization likelihood (Ahmadlou and Adeli, 2011), and phase synchronization (Sauseng and Klimesch, 2008; Gordon et al., 2012; Peraza et al., 2012; Rangaprakash et al., 2013; Serletis et al., 2013) because they are time-frequency and unsymmetrical methods. The value of PDC/DTF is computed from parameters of the multivariate autoregressive (MVAR) model based on the EEG recording. The MVAR model is able to represent interactions among multi-channel EEG signals in the form of linear difference equations. It can be demonstrated that these methods lead to better understanding of unsymmetrical connections between EEG channels (Astolfi, 2007; Wang et al., 2007), and these methods can be effectively used to estimate time- and frequency-varying patterns of functional connectivity between cortical activations (Astolfi, 2006). It has been proven that this method has advantages in improving the reliability and importance of model parameters. The FBN method based on PDC was also applied to an analysis of mental fatigue (Sun et al., 2014), Parkinson’s disease (Tropini et al., 2011), photosensitive generalized epilepsies (Varotto et al., 2012), long-standing vegetative state (Varotto, 2014), and mental rotation task (Zhang, 2009). Blinowska et al. (2013) propose DTF into construction of FBNs to analyze the functional connection in working memory task.

Inspired by Cheung et al. (2009), the optimal model order of the time-varying MVAR model was calculated by ARFIT algorithm (Omidvarnia et al., 2011). Both the time-invariant parameters of the MVAR model and its optimum order *p* were estimated by ARFIT package. A time-varying N-variate AR process of order *p* was expressed as:

(1) $$\left[\begin{array}{c} x_{1}\,\left(n\right)\\ \vdots \\ x_{N}\,\left(n\right) \end{array}\right]=\sum_{r=1}^{p}A_{r}\,\left[\begin{array}{c} x_{1}\,\left(n-r\right)\\ \vdots \\ x_{N}\,\left(n-r\right) \end{array}\right]+\left[\begin{array}{c} w_{1}\,\left(n\right)\\ \vdots \\ w_{N}\,\left(n\right) \end{array}\right]$$

Here, *x* is N-channel signal, *w* is a vector white noise, and the matrices *A*_*r*_ are given by Cheung et al. (2009) and Omidvarnia et al. (2011):

(2) $$A_{r}=\left[\begin{array}{ccc} a_{11}\,\left(r\right) & \cdots & a_{1\,N}\,\left(r\right)\\ \vdots & \ddots & \vdots \\ a_{N\,1}\,\left(r\right) & \cdots & a_{N\,N}\,\left(r\right) \end{array}\right]$$

*r* = 1, 2…, *p* and their elements were estimated using the Yule–Walker equation. The *A* value of PDC can be defined based on the following transformation of the MVAR parameters (*A*_*r*_) to the frequency domain:

(3) $$A\,\left(f\right)=I-\sum_{r=1}^{p}A_{r}\,e^{-i\,2\,\pi \,r\,f}$$

PDC is computed as:

(4) $$\pi_{i\,j}\,\left(f\right)=\frac{A_{i\,j}\,\left(f\right)}{\sqrt{a_{j}^{H}\,\left(f\right)\,a_{j}\,\left(f\right)}}$$

Here, *a*_*j*_ (*f*) indicates the *j*th column of the matrix *A*(*f*), and the superscript *H* is the Hermitian transpose. The value of PDC is between 0 and 1, where a high value in a certain frequency band reflects a directionally linear influence from channel *j* to channel *i* in that band (Channel *i* ← Channel *j*). the DTF was calculated as follows (Kaminski and Blinowska, 1991):

(5) $$v_{i\,j}\,\left(f\right)=\frac{H_{i\,j}\,\left(f\right)}{\sqrt{h_{j}\,\left(f\right)\,h_{i}^{H}\,\left(f\right)}}$$

where *H*(*f*) = *A*^–1^(*f*) is called transfer function matrix, and *h*_*i*_ is the *i*th row of the matrix *H*(*f*).

In the left of the Figure 3, the matrices of PDC or DTF we calculated were 3D [π*_*ij*_* (*f*) and *v*_*ij*_ (*f*) were the elements of the 3D PDC and the DTF matrix, respectively]. The two Ds of PDC or DTF matrix were channels (*i* and *j*: FP1, FP2, F7, F8, F3, F4, O1, and O2), and the third D was *f*: frequency (0–64 Hz). We divided the third D into the following ranges: Delta (δ): 0.5–4.0 Hz, Theta (θ): 4.0–8.0 Hz, Alpha (α): 8.0–14 Hz, Beta (β): 15.0–30.0 Hz, Low gamma (γ): 30.5–50 Hz, All: 0–50 Hz, and then computed the mean value of functional connectivity in different ranges. Hence, the six matrices of different frequency ranges were computed and plotted in Figure 3.

After calculating the adjacent matrix of each Hps and Lps, a brain network can be established (Figures 2C,D). The brain networks were built based on the matrices of PDC or DTF, which were not symmetrical, and the brain networks were directed. The vertices of the brain networks were electrodes. There were at most two edges to link two vertices, one of which is red and the other is blue. Red and blue edges indicate different directions, and the width of the directed edge is proportional to the value of the element in the matrix. In order to make the brain networks look simpler and clearer, only the edges where the PDC or DTF value is higher than the threshold (0.3) were drawn. Weak links and non-significant links (below 0.3) may represent spurious connections, and these links tend to obscure the topology of strong and significant connections, so they are often discarded. Therefore, there was no edge if the PDC or DTF value of a combination was below 0.3.

After drawing the graph, the terms mean degree (D), global clustering coefficient (C), and average characteristic shortest path length (L) (Sporns, 2013) were proposed to quantitatively measure density, connectivity, and even complexity of the networks. The D of a graph is equal to the sum of the connectivity of all the edges. The C is a measure of local structure. The L measures the efficiency of the information transmission in the network. The degree of a node is equal to the number of edges linked to this node and represents centrality, which will help us to find the key node in a graph and will be computed by function (Sporns, 2013):

(6) $$k_{i}=\sum_{j\in N,j\neq i}w_{i,j}$$

where *w*_*i*_,*_*j*_* is the weight of edge from node *i* to node *j*, and *N* is the set of all nodes in the graph. D is computed according to the following equation (Sporns, 2013):

(7) $$D=\frac{\sum_{i\in N}k_{i}}{n}$$

where the *n* is the total number of the nodes. In a weighted network, the C of the graph is computed according to the following equations (Sporns, 2013):

(8) $$t_{i}=\frac{1}{2}\,\sum_{j,h\in N}{\left(w_{i,j}\,w_{i,h}\,w_{j,h}\right)}^{1/3}$$

(9) $$C_{i}=\frac{2\,t_{i}}{k_{i}\,\left(k_{i}-1\right)}$$

(10) $$C=\frac{\sum_{i\in N}c_{i}}{n}$$

where *c*_*i*_ is the clustering coefficient of node *i*. We consider the inverse of the weight of edges as the distance of the edges:

(11) $$d_{u,v}=\frac{1}{w_{u,v}}$$

The characteristic shortest path length from the node *i* to the node *j* is computed according to the following equation (Sporns, 2013):

(12) $$l_{i\,j}=\sum_{a_{u\,v}\in g_{i\to j}}d_{u,v}$$

where the *g*_*i*__→_*_*j*_* is the set of edges that belong to the shortest path from node *i* to node *j*. L is computed according to the following equation (Sporns, 2013):

(13) $$L=\frac{1}{n}\,\sum_{i\notin N}\frac{\sum_{j\in N,j\neq i}l_{i,j}}{n-1}$$

In this study, we applied D, C, and L as the characteristic values for the FBNs based on the two methods, PDC and DTF. There were 36 features in total, because there were six frequency bands and each frequency band has six features. Then, a series of classifiers were used in classification analysis.

There were many classifiers involved to verify the property of the features. To find the best classifier, in our research, the typical classifiers based on various categories including the approach based on distance K Nearest Neighbors classifier (KNN), the approach based on statistical learning theory support vector machines (SVM) with various kernels, and the functional approaches logistic regression (LR) and decision trees (DT) were adopted. Please refer to Chen et al. (2018) for more details about these classifiers. Specifically, we use the Classification Learner toolbox of Matlab R2017b to detect proficiency because it is easy to operate and stable. The inputs of the classifiers were the feature vectors (36 features for one segment) of 3,343 EEG segments, and the targets were the proficiency of the operators (Hps and Lps). The 10-fold cross-validation was used to verify the performance of the classifications. We calculated the accuracy, precision, recall rate, F1-score, and AUC (Kiymik et al., 2004) with the 10-fold cross-validation.

## Results

### Functional Brain Networks

**FIGURE 4:**
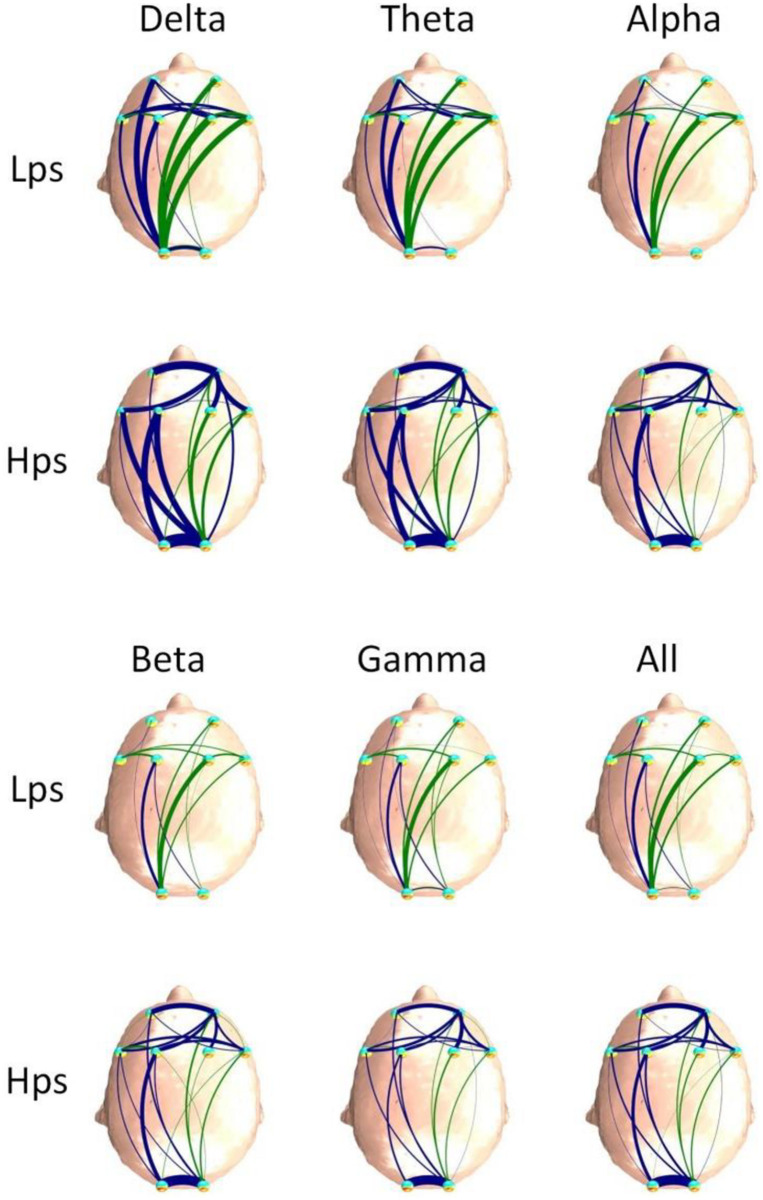
The average FBNs of the two groups and six frequency bands based on the PDC method.

**FIGURE 5:**
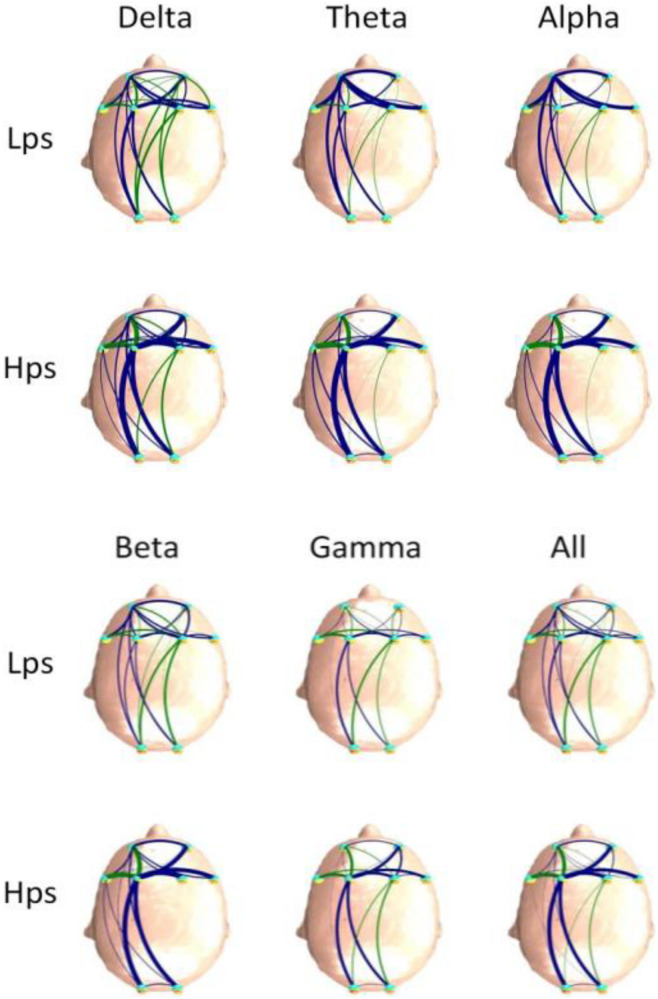
The average FBNs of the two groups and six frequency bands based on the DTF method.

Figures 4, 5 are the FBNs constructed in different frequency bands by the PDC and DTF methods based on average adjacent matrices of all EEG segments from two groups, respectively. The method used to draw these graphs was described above. Through the brain network, the flow direction and flow rate of information is visible. It can be seen that the brain connections of Hps are more than those of Lps for the FBNs based on PDC, and the brain connections of the Hps were stronger than those of Lps for the FBNs based on DTF. The similarity between the two groups is that the connection of the left hemisphere is stronger and more than the connection of the right hemisphere, which is the same as the results obtained by the PDC and DTF methods. Although the graph is easy to observe, it is not convenient for further analysis. Therefore, we introduced graph theory analysis to calculate the D, C, and L of FBN.

### Graph Theory Analysis

**FIGURE 6:**
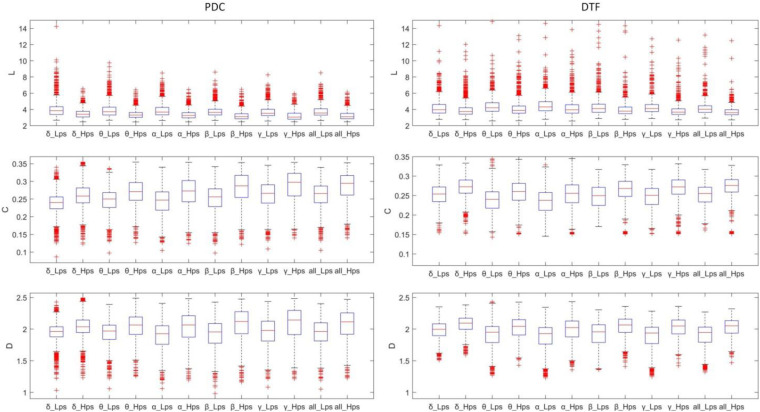
The boxplot of the features (L, C, D) of the different frequency bands and different groups.

Figure 6 shows the boxplots of all graph-theoretic indexes of the operators in the Hps and Lps. For DTF features (especially L), the distribution of values was dispersive, which caused such many abnormal values and such many overlapped values, which means that each feature generated by PDC/DTF and graph theory methods may not be suitable for classification on its own. The comparison of the theoretical indicators of the same graph of different groups generated by PDC and DTF on all the frequency bands follows the same rules: L of the Hps was shorter than Lps, and C of the Hps was higher than Lps. Generally, D of the Hps was higher than Lps (*p* < 0.01, *t*-test, for α and γ with PDC, and for γ with DTF).

Table 1 shows the correlation coefficients between all the features and the target. The calculation of the correlation coefficient is based on the Pearson correlation coefficient, and the targets are the ground truth of Hps and Lps, respectively. The correlation between the feature generated by the PDC method and the target is stronger than the correlation between the feature generated by the DTF method and the target, and the correlations of the L of the PDC were stronger than most other features, while the correlations between the L of the DTF and the target were weak. The correlation coefficient between the features of the high-frequency band (β and γ) and the target is stronger than that of the low-frequency band (δ, θ, and α) (*p* < 0.05, *t*-test). The most target-related features were the L in the β bands.

**TABLE 1:** The correlation coefficient (*r*) and the significance (*p*) between the features and the target.

			δ	θ	α	β	γ	All
PDC	L	*r*	−0.31	−0.35	−0.36	**−0.41**	**−0.37**	**−0.39**
		*p*	6.58e-76	2.96e-98	2.68e-103	**1.40e-133**	**1.62e-110**	**5.25e-124**
	C	*r*	0.32	0.32	0.32	0.34	0.33	0.34
		*p*	3.41e-79	1.63e-82	1.06e-81	1.36e-93	1.02e-83	3.93e-90
	D	*r*	0.26	0.26	0.27	0.31	0.28	0.30
		*p*	5.95e-54	1.14e-52	6.58e-56	6.12e-76	1.80e-62	1.59e-69
DTF	L	*r*	0.04	0.06	0.05	0.05	0.03	0.04
		*p*	1.21e-2	1.23e-3	6.2e-3	4.42e-3	8.09e-2	2.80e-2
	C	*r*	0.28	0.28	0.26	0.27	0.36	0.36
		*p*	4.91e-61	4.35e-59	1.50e-53	1.31e-56	2.61e-105	4.01e-103
	D	*r*	0.29	0.28	0.28	0.31	0.36	0.36
		*p*	2.00e-64	4.75e-60	8.49e-61	8.66e-76	1.80e-103	3.25e-101

*Bold values indicate the best.*

**FIGURE 7:**
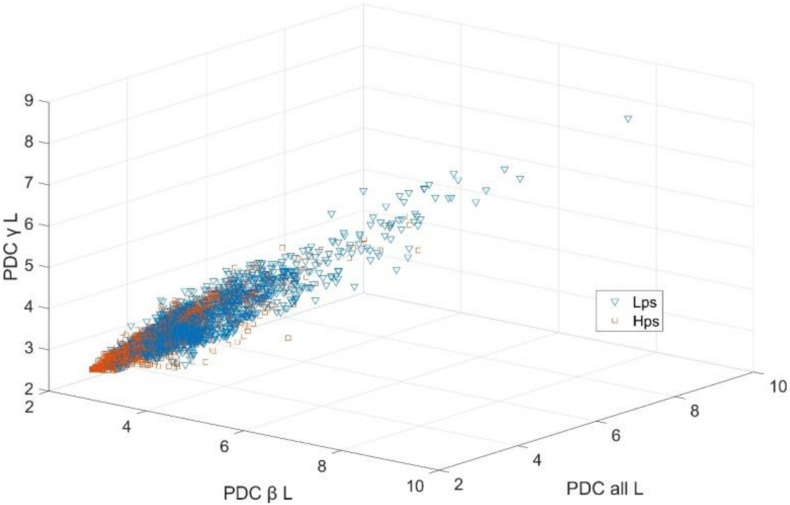
The scatters of all the sample vectors based on the top three features.

Figure 7 shows the scatters of all samples based on the top three features. These three features were the L in β, all, and γ frequency bands. All the three features were created through the PDC method. The red points stand for the feature values of EEG segments in Lps, while the blue points stand for Hps, which means that the values of three indicators of Hps are not always low, and the three indicators of Lps are not always high. There are several points from the two groups that are mixed together, which are difficult to recognize. All 36 features were involved in the feature vectors. The feature vectors corresponding to the EEG segments from two groups are used for classification analysis.

### Classification

Table 2 shows the results of the classification. It can be found that the highest accuracy of the test dataset was up to 94.2% with the KNN classifier. As it can be observed in Table 2, Fine KNN achieves a highest accuracy of 94.2%, a precision of 94.4%, a recall of 94.2%, a highest *F*-score of 94.3%, and an AUC of 0.94, indicating that it has the best classification performance. Although the lowest accuracy of the test dataset was 63.4% with the Quadratic Discriminant classifier, most classifiers could provide a good accuracy above 80%, which means that the features generated by the combination of the PDC/DTF and the graph-theoretic methods were effective and could be used as indexes of the proficiency.

**TABLE 2:** The performance of the different classifiers.

Classifiers	Accuracy (%)	Precision (%)	Recall (%)	*F*-score (%)	AUC
Fine tree	80.7	83.5	79.5	81.5	0.85
Medium tree	76.3	81.4	74.5	77.8	0.82
Coarse tree	72.5	78.3	70.8	74.4	0.76
Linear discriminant	78.7	75.8	81.1	78.3	0.87
Quadratic discriminant	63.4	98.4	58.3	73.2	0.89
Logistic regression	80.5	80.3	81.1	80.7	0.88
Linear SVM	80.0	79.2	81.1	80.1	0.88
Quadratic SVM	89.1	87.1	91.0	89.0	0.96
Cubic SVM	93.9	93.8	94.2	94.0	0.97
Fine Gaussian SVM	93.5	94.6	92.7	93.7	0.98
Medium Gaussian SVM	84.3	80.9	87.3	84.0	0.92
Coarse Gaussian SVM	75.5	77.7	75.0	76.3	0.84
**Fine KNN**	**94.2**	**94.4**	**94.2**	**94.3**	**0.94**
Medium KNN	87.8	89.7	86.9	88.2	0.95
Coarse KNN	77.5	75.9	79.1	77.4	0.85
Cosine KNN	87.2	86.5	88.1	87.3	0.95
Cubic KNN	87.0	88.7	86.1	87.4	0.94
Weighted KNN	92.6	91.8	93.6	92.7	0.98
Boosted trees	82.9	82.8	83.4	83.1	0.92
Bagged trees	90.8	91.9	90.2	91.0	0.97
Subspace discriminant	77.1	75.1	78.9	77.0	0.86
Subspace KNN	93.7	93.7	94.0	93.8	0.98
RUSBoosted trees	77.2	82.6	75.1	78.7	0.85

*Bold values indicate the best.*

## Discussion

In order to recover the brain executive process of operators with tacit knowledge, a method that combines brain cognition process with industrial controlling process was proposed and used to detect the proficiency of operators. The graph theory analysis was used to measure the brain networks of the operators based on EEG. The feature set containing D, C, and L of the two methods in all the frequency bands was applied to the classification analysis. The highest accuracy among all classifiers is up to 94.2% (see Table 2).

Compared with our study, all previous studies in FBNs focused on mental diseases, cognitive tasks, and different mental state. As in most industrial processes, operators also play a vital role in the mineral grinding process, but few people study the mental state of industrial operators based on EEG signals. In this study, FBN was applied to the industrial process to detect the proficiency of operators. Because the operation of mineral grinding was a complex process containing various kinds of cognitive process instead of a simple singular cognitive task, it was possible and reasonable that the results, which are the indicators of brain networks based on PDC/DTF of Hps, are higher than Lps. The results are in conflict with the results of Deeny et al. (2009). They applied the EEG coherence method to the analysis of the functional couples in the visuo-motor tasks, and the result of their study is that compared with novices, experts usually show lower coherence.

In this study, the global indicators D, L, and C based on graph theory were used to reveal the difference in brain networks between Hps and Lps, which was different from other research (Barry et al., 2002; Babiloni, 2011; Del Percio, 2011). They used the coherence method to analyze the difference of functional cortical connections between two groups of different states, and paid attention to analyze specific connections of cortical regions within each of the δ, θ, α, and β bands, to reveal details of the activities of the specific cortical region. Their method was helpful to reveal the function of specific brain regions, but compared with such research focusing on specific connections, the global feature was more stable. Because different subjects have differences in the location of neural activity and brain function areas, particularly based on the EEG method, the local features of the brain networks (such as the PDC and DTF value between the signals collected from two specific regions) are instable. Our results suggested that the global clustering coefficient (C) and average of the brain networks of Hps were higher than those of Lps, while the average characteristic shortest path length (L) was opposite. These parameters indicate that the network of Hps is a small-world network, in which the speed of information transmission is faster.

There are some limitations in this study. The PDC/DTF methods are linear methods and can be extended to assess but not reveal the non-linear characteristic of the cortical activations. Due to the intrinsic non-linearity of neuronal activity (Stam, 2005; Adeli et al., 2008; Vejmelka et al., 2010), the non-linear method like non-linear Granger causality (He et al., 2014) will be used in our study in the future. Moreover, although some cross-validation and performance analysis have been provided, the sample size of 20 participants still needs to be increased. Another limitation of the study is that no evaluation of the connectivity before the experiment was performed. Moreover, the reported results were valid only as far as the EEG was recorded during the sessions at the simulator. Based on the limitations discussed above, the presumed equivalence of the neural features in the two groups will be evaluated before training and whether different hallmarks of connectivity between the two groups can be found also in the EEG recordings during other tasks will be explored in our future study directions.

According to the current result of this study, the FBN method based on PDC/DTF could assist the analysis of proficiency of the operators. Based on the analysis of the operator’s brain network characteristics, we established the relationship between the operational control proficiency and the EEG characteristics, so the industrial control process information is linked to the human brain cognitive characteristics. Additionally, we will apply this detective system to the real-time grinding process and send the proficiency of the operator as the prompt message to the monitor in front of the operator in the future.

## Conclusion

In this study, we applied PDC and DTF to build the FBN method to extract the features for the detection of proficiency of operators in the mineral grinding process. Furthermore, we revealed the effect of tacit knowledge on the structure of functional neural system during the task. The results suggested that the mean degree (D), global clustering coefficient (C), and average of the brain networks of Hps were higher than those of Lps, while the average characteristic shortest path length (L) was the opposite (*p* < 0.01, *t*-test). The feature vectors calculated based on the brain networks built *via* the PDC and DTF method can be used as the input of the classifiers to indirectly detect the proficiency of operators, and the accuracy of the classification in the paper was up to 94.2%.

## Data Availability Statement

The raw data supporting the conclusions of this article will be made available by the authors, without undue reservation.

## Ethics Statement

The studies involving human participants were reviewed and approved by the Institutional Review Board at NEU. The patients/participants provided their written informed consent to participate in this study.

## Author Contributions

TZ conceived the methodology and developed the algorithm. CH designed and performed the experiment. CH, TZ, and JC analyzed the data and wrote the manuscript. HW and EH. finished editing and proofreading. All authors have read and agreed to the published version of the manuscript.

## Conflict of Interest

The authors declare that the research was conducted in the absence of any commercial or financial relationships that could be construed as a potential conflict of interest.
